# An exploration into physician and surgeon data sensemaking: a qualitative systematic review using thematic synthesis

**DOI:** 10.1186/s12911-022-01997-1

**Published:** 2022-09-28

**Authors:** Emma Whitelock-Wainwright, Jia Wei Koh, Alexander Whitelock-Wainwright, Stella Talic, David Rankin, Dragan Gašević

**Affiliations:** 1 Faculty of Information Technology, Centre for Learning Analytics (CoLAM), Monash University, Melbourne, Australia; 2 School of Public Health and Preventative Medicine, Monash University, Melbourne, Australia; 3 Clinical Governance and Informatics, Cabrini Health, Melbourne, Australia; 4 Practice Analytics, DHCRC, Sydney, Australia

**Keywords:** Continued professional development, Digital health, Lifelong learning, Performance reflection, Physicians and surgeons, Practice analytics, Professional learning, Sensemaking

## Abstract

Providing electronic health data to medical practitioners to reflect on their performance can lead to improved clinical performance and quality of care. Understanding the *sensemaking* process that is enacted when practitioners are presented with such data is vital to ensure an improvement in performance. Thus, the primary objective of this research was to explore physician and surgeon sensemaking when presented with electronic health data associated with their clinical performance. A systematic literature review was conducted to analyse qualitative research that explored physicians and surgeons experiences with electronic health data associated with their clinical performance published between January 2010 and March 2022. Included articles were assessed for quality, thematically synthesised, and discussed from the perspective of sensemaking. The initial search strategy for this review returned 8,829 articles that were screened at title and abstract level. Subsequent screening found 11 articles that met the eligibility criteria and were retained for analyses. Two articles met all of the standards within the chosen quality assessment (Standards for Reporting Qualitative Research, SRQR). Thematic synthesis generated five overarching themes: data communication, performance reflection, infrastructure, data quality, and risks. The confidence of such findings is reported using CERQual (Confidence in the Evidence from Reviews of Qualitative research). The way the data is communicated can impact sensemaking which has implications on what is learned and has impact on future performance. Many factors including data accuracy, validity, infrastructure, culture can also impact sensemaking and have ramifications on future practice. Providing data in order to support performance reflection is not without risks, both behavioural and affective. The latter of which can impact the practitioner’s ability to effectively make sense of the data. An important consideration when data is presented with the intent to improve performance.

*Registration* This systematic review was registered with Prospero, registration number: CRD42020197392.

## Background

Electronic health data is leveraged for many secondary purposes in healthcare, namely clinical decision making [1] and quality improvement [2, 3]. Less research has explored how such data can support lifelong learning in healthcare, and more specifically, how it can support a medical practitioner’s *continuing professional development* (CPD). Janssen et al. [4] highlight this notable research gap. They stress both the opportunity to provide actionable data to practitioners to individually reflect on their performance, and the subsequent positive impact this could have on health outcomes. Research that explores this is within scope of the emerging area of *practice analytics* [4]. Practice analytics explores how such data can be used to facilitate performance reflection, support CPD, and thus lead to improvement in the quality of care. A crucial component of which is ensuring the data is meaningful, and for this we argue for an exploration into a practitioner data *sensemaking*.

### Continuing professional development (CPD)

CPD includes activities that are tailored to individual learners that allow them to build upon existing knowledge to ensure optimal competence [5]. It is an ongoing process of learning through self-evaluation and self-reflection, which leads to behavioural change and measurable improvement in health outcomes [5]. Many activities constitute towards CPD, inclusive of e-Portfolios, performance reflection, and demonstrations of competence [6]. Such activities are central to maintaining and developing clinical skills, and promote safe patient-centered care. Given this, in countries such as Australia, Canada, and United Kingdom (UK), practitioners must demonstrate a variety of development activities, in order to retain their certification to practice [7–9]. Lockyer et al. [10] highlight the key role that performance data plays within such activities, and this is further emphasised by the inclusion of digital, data-informed CPD within professional frameworks [8, 11–14]. A notable example is within Australia, where from January 2023, practitioners are expected to spend a minimum of 25 hours per year reviewing data associated with their clinical performance and outcomes [8]. Practitioners who review such data report greater intentions to improve [15], increased performance awareness and compliance [16], and improved patient outcomes [16].

Data within health information systems can form the basis of feedback that leads to practice improvement [17]. What is more, many different data-informed initiatives can lead to performance reflection and constitute towards CPD. Lockyer et al. [6] found that practitioners *“used and reflected on many non-formal non-explicit sources of data provided by their professional colleagues, patients, and the educational resources”* (p.e119). This highlights that many different data sources can initiate self-reflection or a review of performance, which include formal processes such as audit and feedback [18], web-based audits [19], or performance dashboards/reporting [20]. This is in addition to data that practitioners may access directly themselves (e.g. benchmarking via clinical registries [21], or accessing aggregated data via learning health systems [22]). However, the latter approach is much less common. This is emphasised by Sockalingam et al. [23] who highlight that data associated with practice can support education; however, even when available, is not universally used. This is despite calls for both practitioners to retain accountability of their own lifelong learning thorough reviewing clinical performance data, identifying areas for improvement, and aligning future development activities to address any shortfalls [10]. A key focus of practice analytics research.

### Practice analytics

The emerging research area of practice analytics explores how data in healthcare can be effectively leveraged to improve the quality of care. Specifically, how data can support performance reflection and CPD [4]. This research is needed for many reasons. First, to complement the emphasis that professional development frameworks place upon performance data reflection [8]. Second, to satisfy practitioners needs and increased interest in accessing data to review their own performance for the purpose of development and learning [24].

For many practitioners, independently reflecting on performance and outcome data may be a new concept. Integrating self-directed reflection into their routine may be novel, and beyond this, the process of self-assessment is notoriously complex, with many different cognitive processes at play [25, 26]. Notably, Sargeant et al. [26] highlight the complexities surrounding how data is understood, used, and the conditions that influence such process (e.g. emotions, environment, tensions). For these reasons, we argue for data and tools that are grounded in the needs of practitioners, to ensure that data is presented in a way that is actionable, meaningful, and leads to improved practice [27]. Practice analytics addresses this by focusing on practitioners to understand what indicators are meaningful, how the data should be presented, and how practitioners *make sense* of such data [4]. Here we begin the focus on the last of such concepts – how practitioners make sense of data; that is, the *sensemaking* process.

### Sensemaking

Sensemaking is defined as *“a process, prompted by violated expectations, that involves attending to and bracketing cues in the environment, creating intersubjective meaning through cycles of interpretation and action, and thereby enacting a more ordered environment from which further cues can be drawn”* (p.67) [28]. Succinctly, it is a process initiated when an individual is presented with a situation that is novel or unexpected in order to assign meaning to it and restore sense [28]. Whilst sensemaking includes interpretation, sensemaking is considered more individualistic as meaning is created through a function of interpretation *and* individual knowledge, prior experiences, and other situational factors [29]. Research into sensemaking spans many different contexts, inclusive of organisational psychology [30], human-computer interaction [31], learning analytics [32], and information science [33].

**Fig. 1:**
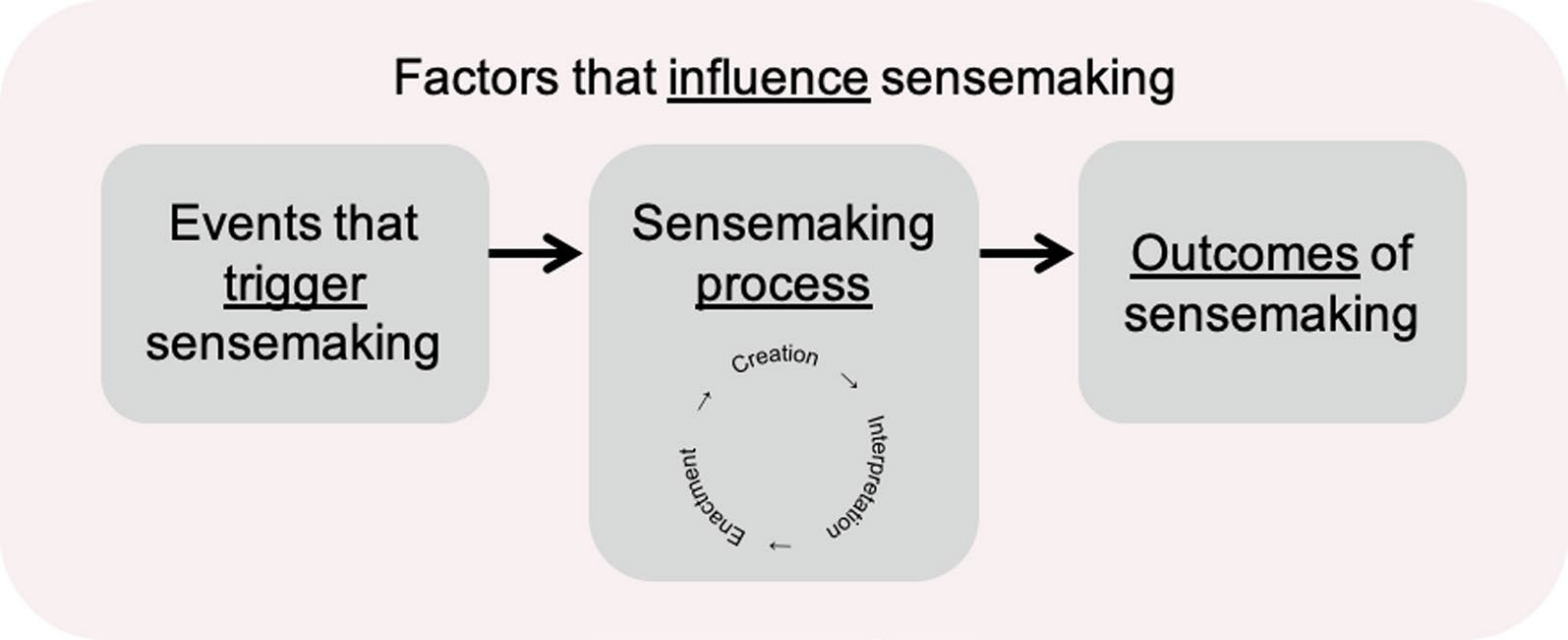
A figurative adaptation of the “major constituents of the sensemaking perspective” presented by Sandberg and Tsoukas [34]

Sandberg and Tsoukas [34] present the “major constituents of the sensemaking perspective” based on an extensive aggregation of research and literature with the area. They outline (i) events that trigger sensemaking, (ii) the process of sensemaking, (iii) factors that influence sensemaking, and (iv) the outcomes of sensemaking. A visual representation, adapted from Sandberg and Tsoukas [34], can be found in Fig. 1 and a written summary is below.

(i) * Events that trigger sensemaking:* Sensemaking is initiated in order to restore sense when it is interrupted. This can be initiated by both planned or unplanned events [34].
(ii) *The process of sensemaking:* The process of restoring sense involves many different smaller processes that are considered retrospective [30, 34, 35]. They are considered retrospective because they rely on an individual’s past experiences to make sense of the “present” experience, and include creation, interpretation, and enactment [30, 34]. In summary, individuals *“first* ***create*** *what they subsequently focus on for* ***interpretation*** *and* ***act*** *on those interpretations; the cycle is ongoing”* (p.S14, bold added for emphasis on processes) [34]
(iii) *Factors that influence sensemaking:* All of the above can be affected by many factors, including context, emotion, and technology [34, 36].
(iv) *Outcomes of sensemaking:* The outcome of sensemaking is that sense is restored [34]. The above processes and factors require consideration when data is presented to ensure that the outcome is meaningful for performance reflection and improvement.

It is important to consider the sensemaking process that is enacted when data is presented to medical practitioners for many reasons. First, users who experience challenges when making sense of data struggle to get actionable information that can translate into behavioural change [37]. Second, the process of sensemaking is individualistic, and what is meaningful to one person may differ from another [38]. Finally, many of the factors that have already been shown to influence sensemaking (e.g. context, emotion, and technology – highlighted above) may be at play when the data is presented to practitioners.

## Rationale

Research surrounding health data sensemaking makes a critical shift within the field of health communication by *“humanising data”*, not *“data-fying humans”* [39]. However, there remains no exploration into the sensemaking process that is enacted when medical practitioners interact with electronic health data associated with their clinical performance and outcomes. This is despite calls to explore how individuals think about and make sense of data associated with their clinical practice [7], and also the increasing amount of data that practitioners interact with (e.g. the accelerated implementation of electronic health/medical records [40]. As such, this work not only has implications for using data to support performance reflection and development, it can offer a better understanding of medical practitioners interactions with other routine data (e.g. electronic health/medical records).

This review is the first exploration into sensemaking in the context of practice analytics. However, given the variety of roles within healthcare, the scope of this review was limited to exploring the process of sensemaking within physicians and surgeons as defined by the Medical Board of Australia [41] only.

## Objective

The primary objective of this research is to review and synthesise literature that has qualitatively explored physician and surgeon experiences with data associated with their clinical performance. Such synthesis will be used to provide insights into the sensemaking process itself, and also identify any gaps in knowledge and implications for sharing data in healthcare to support practitioner development.

## Methods

This review followed the Preferred Reporting Items for Systematic Reviews and Meta-Analyses guidelines (PRISMA) [42].

### Eligibility criteria

Table 1 presents the review eligibility criteria, which was developed using the SPIDER framework (*S*ample, *P*henomenon of *I*nterest, *D*esign, *E*valuation, *R*esearch Type) [43]. This was inclusive of articles published between 1 January 2010 and 10 March 2022 and in English.

**Table 1:** Eligibility criteria developed using the SPIDER framework

SPIDER	Eligibility criteria
Sample	Physicians and surgeons who practice in roles recognised by the Medical Board of Australia [41] were included. For international clarity, physicians included specialist doctors, a full break down of which is provided by the Medical Board of Australia [41].
	All other medical or health practitioners were excluded for example nurses, physical therapists, or pharmacists.
	The articles was excluded if the sample was combined or the specific role was unclear, for example “health professional”.
	Physicians or surgeons were fully trained. Those completing internships or medical residency programs were excluded.
Phenomenon of Interest	Clinical performance data or feedback that had been derived from an electronic source, for example electronic health record or patient administration system, were included.
	Articles that did not provide the above were excluded, this included exploration into the *prospect* of using data in this way.
Design	All qualitative research designs were included as they all provided insights into the experiences with clinical performance data or feedback.
	Mixed-methods research designs were included however only the qualitative results were included for analysis. For example, open text responses, to an otherwise quantitative survey, were included.
Evaluation	The sensemaking process was evaluated.
	As no articles explored this directly, included articles were synthesised and reviewed against existing sensemaking literature to address the research objectives.
Research Type	Both qualitative and mixed-methods research were included however only the qualitative aspects will be analysed.
	Quantitative research was excluded. This is because no research has explored the sensemaking process when interacting with clinical performance data or feedback, therefore no inferences could be made from quantitative research that does not explore this.

### Information sources

On 29 October 2020, EWW searched four databases using the Ovid platform: Extended MEDLINE, EMBASE, Cochrane Central Register of Controlled Trials, and PsychInfo. Only peer reviewed journal articles were included, and no grey literature was searched. EWW used two additional snowballing techniques to search for articles. This made use of the final set of articles. First, they screened reference lists for potential inclusions. Second, they used backward and forward citation searching using Google Scholar. Identified articles were subject to screening. Inter-library requests for selected full-text articles occurred when the text was unavailable through the Monash University library. Ahead of publication, the full search was re-run on 10 March 2022, three further articles were found that met the aforementioned criteria.

### Search strategy

Additional file 1: Appendices A1–A4 present the full line-by-line search strategy for each database. Each search strategy used a combination of Medical Subject Headings (MeSH) and free text words, structured using the SPIDER framework (Table 1). The strategy used the Boolean term “OR” to combine words associated with the Phenomenon of Interest, and then combined these with terms associated with the Sample and Research type using the Boolean term “AND”.

To develop the search strategy, the research team consulted with a health-subject librarian. Search terms used within the “Sample” section (Additional file 1: Appendices A1–A4) aligned with physician and surgeon specialities outlined by the Medical Board of Australia [41] whilst accounting for international spellings and naming conventions. The search terms for “Phenomenon of Interest” and “Research Design” (Additional file 1: Appendices A1–A4) were developed using three known relevant articles [44–46] to identify potential MeSH terms and free text search words. Construction of these search terms followed an iterative process of testing, expanding, and refining. To assess the validity of the search strategy, the researchers checked that the initial articles remained within the search.

Initially we restricted the search strategy to articles published between 1 January 2010 and 29 October 2020 and in English. Ahead of publication submission, and to remain current, the search was then re-run to find articles that were published between 1 January 2010 and 10 March 2022.

### Selection process

Two researchers (EWW & JWK) completed the title and abstract screening both independently and blindly. Throughout this process, they met regularly to resolve any conflicts by reaching a consensus. The researchers repeated this process for the full-text screening.

### Data items & collection process

One researcher (EWW) extracted the data from the final articles. To remain objective, *“... all of the text labelled as ‘results’ or ‘findings’ in study reports ...”* (p.4) [47] were extracted for analysis. Two researchers (EWW & AWW) also extracted publication year, country, research design, research aim(s), study setting, sampling approach, details about how the data was disseminated, data collection methods, and type of qualitative analysis conducted.

### Assessment of methodological quality

To assess quality, the researchers used the Standards for Reporting Qualitative Research (SRQR) [48]. Selection of this quality measure was appropriate, as the researchers only synthesised qualitative elements.

Two researchers (EWW & AWW) assessed each included article against the SRQR criteria, calculating a total quality score per article. This score represented the proportion of standards that the article met. No exclusions resulted from this step.

### Synthesis methods

Thematic synthesis [47] was used to synthesise the results of all articles that reached the stage of full-text analysis. To ensure reliability, articles were independently line by line coded by two researchers (EWW & AWW) who met at intervals to review codes and discuss emerging themes. The codes and emerging themes within the articles were then iteratively reviewed to generate the final set of themes. All articles were manually coded, no specific software was used for this process.

This process generated descriptive themes, meaning that they remained similar to that of the original work [47]. This approach was taken for a few reasons. First, there are many different sensemaking perspectives and theories, not “one” main theory that could guide the deduction of analytical themes. In addition, given that this is the first exploration into sensemaking in this context, we deemed it inappropriate to select one of these perspectives and enforce this to and entirely new context. Second, descriptive themes were clearer and more replicable in this case, particularly to those who are less familiar with sensemaking. This allowed us to explicitly link each theme to the many different aspects of sensemaking research in the discussion to address the research objective.

### Assessment of confidence in qualitative findings

In order to assess the confidence of the qualitative findings, CERQual (Confidence in the Evidence from Reviews of Qualitative research) [49, 50] was used. This allowed for a systematic and transparent assessment of confidence in the findings through the assessment of (i) methodological limitations [51], (ii) coherence [52], (iii) adequacy of data [53], and (iv) relevance [54] for each sub-theme. This was done on a sub-theme level as this is the level of detail that is integrated into the sensemaking discussion in order to address the research objective. A summary of each CERQual component is provided below.

(i) *Methodological limitations* assessed the design or conduct of the original articles that contributed to that finding [51].
(ii) *Coherence* evaluated how substantially the finding within the review aligned with the original article [52]
(iii) * Adequacy of data* assessed how much data existed to support such finding [53].
(iv) * Relevance* assessed how applicable the finding was to the context [54].

Upon reviewing each component, the findings were given an confidence assessment of either high, moderate, low, or very low confidence. This was conducted by two researchers (EWW & AWW).

## Results

### Study selection

**Fig. 2:**
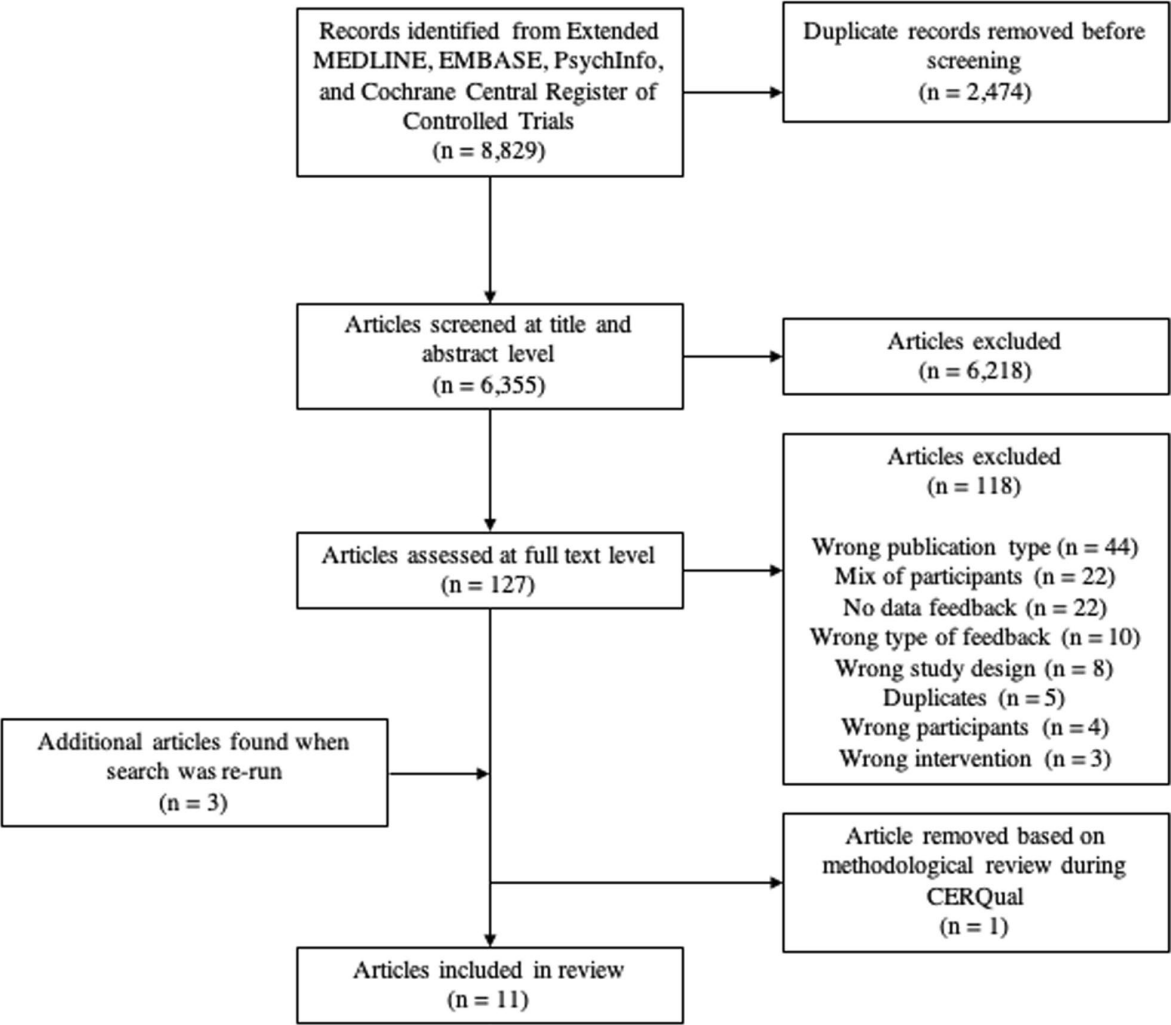
PRISMA flow diagram outlining the article selection and screening process

The initial search returned 8,829 articles, dropping to 6,335 for title and abstract screening with the removal of duplicates. Researchers screened 127 articles at a full-text level. A total of 118 articles were excluded at this stage, the reasons for exclusion are highlighted in Fig. 2. To clarify three of these reasons that are more ambiguous, first, “wrong publication type” included results that were not full text articles, for example, abstracts for conference presentations or posters. Second, a “mix of participants” included results that either, grouped their sample more generally (e.g. health professionals), or did not separate out the results of physicians or surgeons (e.g. results were synthesised to include other professions such as nurses). Finally, “wrong type of feedback” included articles where feedback was not on *clinical* performance but instead an alternative measures such as communication performance. Three articles were subsequently found when the search was re-run ahead of publication submission.

A total of 12 articles initially met the inclusion criteria [44–46, 55–63]. Researchers then found these 12 articles using Google Scholar and used backward and forward citation which resulted in no further articles for inclusion. Checking reference lists of all included articles also resulted in no further inclusions. One article [46] was later removed during the CERQual process. This was because, upon critical review, the researchers felt that the detail provided in relation to the methodological approach (data simulation), did not clearly align with the Phenomenon of Interest (see Table 1) of this review. As such, 11 articles were included within the full review [44, 45, 55–63]. Figure 2 summarises both the article selection and screening process.

### Study characteristics

Tables 2 and 3 present the characteristics of the studies included within this review. The majority of studies took place in Canada (64%), and the remainder were from the United States of America (USA). No studies specifically explored sensemaking, instead their aims included exploring experiences, perceptions, behaviours, evaluating processes, barriers, and enablers of performance data reporting. To which the data was collected and disseminated in a few different ways, the most common was audit & feedback [44, 45, 55–57, 60–62], followed by surgeon-specific performance reports [59, 63], and one study was part of a quality improvement activity [58]. A variety of different analyses were used: a form of thematic analysis [55–59], grounded theory [45, 60, 62], framework approaches [44, 61], and a constant comparative method [63].

**Table 2:** Characteristics and quality score of included studies

Article	Quality score	Country	Research design	Aim	Study setting	Intervention (Data dissemination process)	Sampling Approach	Data collection methods	Qualitative analysis approach
Barber et al. [55]	95.2%	Canada	Mixed-methods	1. To describe both the operationalisation and reporting of practice performance. 2. To describe rheumatologists’ experiences with reports and group feedback.	Rheumatology Clinic	Audit & Feedback	Rheumatologists who had received individual reports on their practice were invited to complete a survey and interview.	A survey was used to explore report acceptability and usefulness. Semi-structured interviews were conducted to elaborate on experiences.	Thematic Analysis
Cooke et al. [56]	90.5%	Canada	Qualitative	1. To explore physicians behaviours during a group audit and feedback session. 2. To explore how sessions lead to practice change and implementation discussions.	Cumming School of Medicine, University of Calgary	Audit & Feedback	All group audit and feedback sessions that took place between January 2015 and January 2016 were used.	Recorded group audit and feedback sessions were transcribed for analysis.	Thematic Analysis
Desveaux et al. [57]	100%	Canada	Qualitative	1. To understand cognitive engagement when physicians engage with audit and feedback. 2. To explore how to close the gap between intention and action.	Primary care organization (six clinics)	Audit & Feedback	Physicians were invited to take part if they submitted a self-reflection task after intervention. Told about the research in a staff meeting and follow up reminder emails were used. Recruited until saturation was reached (June-September 2018).	Audio recorded, qualitative semi-structured interviews that were transcribed verbatim were used in conjunction with qualitative data from the self-reflection task.	Thematic Analysis
Eden et al. [58]	71.4%	USA	Qualitative	1. To evaluate family physicians’ perceptions towards performance and peer comparison feedback.	Online survey	Quality Improvement Performance Feedback	Physicians who supplied free-text comments in an online survey (2004-2014).	Three open-ended feedback questions covering how to improve Performance in Practice Modules.	“grounded approach to” Thematic Analysis
Ivanovic et al. [59]	71.4%	Canada	Mixed-methods	1. To create individualised surgeon performance reports. 2. To implement a surgery quality improvement program. 3. To understand surgeons’ perceptions towards the above.	Hospital	Surgeon-Specific Outcome Reports	Six surgeons within the Division of Thoracic Surgery. No additional sampling information was provided.	Interviews to identify facilitators and barriers of using surgeon-specific outcome reports and seminars.	Thematic Analysis

### Assessment of methodological quality summary

Tables 2 and 3 present the overall quality score for each article. A full article-level breakdown of these scores is found in Additional file 1: Appendix B1. Table 4 shows the adherence to each of the individual standards within the SRQR; two [57, 61] articles met all the standards.

**Table 3:** Continuation of characteristics and quality score of included studies

Article	Quality score	Country	Research design	Aim	Study setting	Intervention (Data dissemination process)	Sampling approach	Data collection methods	Qualitative analysis approach
Ivers et al. [44]	85.7%	Canada	Qualitative	1. To understand the perceived usefulness of external feedback. 2. To understand perceived barriers and facilitators to using this to improve practice.	Multidisciplinary primary care practices	Audit & Feedback	Stratified purposive and snowball sampling was used to recruit family physicians. Sampling ensured variation in sex, experience, and baseline performance.	Semi-structured interviews were conducted. The interview protocol was piloted and adapted as the interviews were conducted.	Framework Approach
Kamhawy et al. [60]	95.2%	Canada	Qualitative	1. To understand physician experiences with practice data. 2. To develop a model that outlines how physicians interact with practice data.	Across seven practices (emergency physicians)	Audit & Feedback	Intentional sampling based on constructivist grounded theory to ensure representation of different practice characteristics.	Interviews were conducted and the guide was based on a prior survey. Interviews were audio-recorded and transcribed.	Constructivist Grounded Theory
Laur et al. [61]	100%	Canada	Qualitative	1. To explore how and why physicians change their prescribing behaviour in response to receiving an audit and feedback report.	Nursing homes	Audit & Feedback	All physicians who had received the report were eligible to take part. A statement in the report invited individuals to take part. A further recruitment email was then needed to recruit physicians.	Interview questions were guided by a predetermined theoretical framework. Interviews were audio-recorded and transcribed verbatim.	Framework Analysis
Payne and Hysong [45]	90.5%	USA	Qualitative	1. To determine which elements of the audit and feedback process influence acceptance. 2. To explore post-feedback actions.	Veterans Affairs Medical Centres	Audit & Feedback	Random sample of full time primary care physicians who met the inclusion criteria.	Semi-structured interviews were used using a predetermined interview protocol.	Grounded Theory
Szymczak et al. [62]	95.2%	USA	Qualitative	1.To explore pediatricians’ experiences of an antimicrobial stewardship intervention and antibiotic overuse	Primary care practices	Audit & Feedback	Respondents were invited if they met the eligibility criteria. Recruitment occurred via email. Recruitment continued until saturation of themes was reached.	Open-ended, semi-structured interviews were conducted. The interview protocol was developed based on a literature review and team discussions.	Grounded Theory
Yi et al. [63]	95.2%	USA	Qualitative	1. To evaluate the use of surgeon-specific reporting in surgery. 2. To assess if the reporting enables performance self-assessment and to identify barriers.	Hospital	Surgeon-specific Performance Reports	The surgeon had to meet a series of eligibility criteria regarding surgery volume. Surgeons were invited to take part via email.	Semi-structured interviews were conducted. The interview protocol was designed to assess the usefulness of reports and overall impressions.	Constant Comparative Method

Of the 21 standards within the SRQR, 12 were met across all articles (Table 4) [44, 45, 55–63]. This included the requirements associated with research questions, ethical considerations, data collection and analysis methods, and all elements of the discussion. 10 [44, 45, 55–58, 60–63] of the 11 articles met all requirements within the “Results/findings” section of the SRQR (Table 4).

**Table 4:** A list of each of the standards within the SRQR and both the number and percentage of articles that met these standards

Standard taken directly from the SRQR	Number of articles that met this standard	Percentage of articles that met this standard
Title and abstract		
S1: Title	3	27%
S2: Abstract	11	100%
Introduction		
S3: Problem formulation	11	100%
S4: Purpose or research question	11	100%
Methods		
S5: Qualitative approach and research paradigm	11	100%
S6: Researcher characteristics and reflexivity	7	64%
S7: Context	10	91%
S8: Sampling strategy	9	82%
S9: Ethical issues pertaining to human subjects	11	100%
S10: Data collection methods	11	100%
S11: Data collection instruments and technologies	11	100%
S12: Units of study	10	91%
S13: Data processing	11	100%
S14: Data analysis	11	100%
S15: Techniques to enhance trustworthiness	9	82%
Results/findings		
S16: Synthesis and interpretation	11	100%
S17: Links to empirical data	10	91%
Discussion		
S18: Integration with prior work, implications, transferability, and contribution(s) to the field	11	100%
S19: Limitations	11	100%
Other		
S20: Conflicts of interest	10	91%
S21: Funding	8	73%

The standards that were met less frequently were the requirements for the “Title” (27% of articles) and “Researcher characteristics and reflexively” (64% of articles). In relation to the title, eight articles failed to identify the study design, approach, or collection methods in their title. In relation to the researcher characteristics and reflexively, four articles failed to identify or acknowledge this.

### Assessment of confidence in qualitative findings summary

An overview of the assessment of confidence in qualitative findings grouped by sub-theme can be found in Additional file 1: Appendices B2–B3. As referenced earlier, one study [46] was removed from the review as a result of such process. As a result of omitting the study, the results were iteratively reviewed. The removal of the study had no impact on the synthesised themes as the findings were well established across the other studies.

Overall, the confidence in the sub-themes within the review ranged from low to high confidence, the majority were classed as moderate confidence (seven sub-themes). The findings are presented in Additional file 1: Appendices B2–B3, and the salient concerns for each component of CERQual are summarised below.

*Methodological limitations* As also highlighted by the SRQR, the majority of the studies lacked comment on researcher reflexivity. Other common methodological concerns included the use of secondary analysis that lacked alignment with research aims, and lack of detail surrounding sampling strategies and approaches. However, overall eight studies had either no, or only minor, concerns raised.

*Coherence* There were very few concerns raised in relation to coherence. All sub-themes had either no, or only minor, concerns highlighted. The minor concerns that were raised were in relation to less focus being placed on such finding within the original study.

*Adequacy of data* In general, there were large amounts of data to support each finding. Only one sub-theme was labelled to have moderate adequacy concerns, the remainder had minor to no concerns. This is also emphasised by the themes (and sub-themes) remaining both well-established and consistent even after the removal of a study.

*Relevance* Likely as a result of the search strategy, the majority of the included studies had moderate concerns in regards to relevance (eight had moderate concerns and one high concern). The main concerns centered around the broader aims of the studies as they had a wide variety of different objectives. Given that no research has explored sensemaking in this context, any qualitative research design that provided insights into such process were included (see Table 1), and as a result the studies were broad in scope and relevance.

In addition to the broad aims, some studies were also highly specific to certain contexts or speciality groups, meaning they were less relevant to the general population. For example, whilst some studies designs leveraged more widely relevant data sets (e.g. administrative data or the electronic medical record), some used locally developed data sources likely only relevant to one specialist group or context.

### Results of synthesis

Thematic synthesis of the included articles generated five overarching themes, each with sub-themes. Table 5 outlines all of the themes and sub-themes, and highlights the articles that were associated with each theme as a result of thematic synthesis. What follows is a summary of each of theme. Quotes to support each of these themes can be found in Additional file 1: Appendices C1–C5. Such quotes were lifted directly from their original source and due to differences in how authors structured their results, they include a combination of both the authors qualitative interpretation and quotes that were used to support these.

**Table 5:** A list of the themes, sub-themes, and corresponding articles generated by thematic synthesis

	Barber et al. [55]	Cooke et al. [56]	Desveaux et al. [57]	Eden et al. [58]	Ivanovic et al. [59]	Ivers et al. [44]	Kamhawy, Chan and Mondoux [60]	Laur et al. [61]	Payne and Hysong [45]	Szymczak et al. [62]	Yi et al. [63]
Theme 1: Data Communication											
Presentation	X	–	–	X	–	X	–	–	X	–	X
Interpretation	X	X	X	–	–	X	X	–	X	X	X
Theme 2: Performance Reflection											
Attribution	X	–	X	X	–	–	X	X	X	–	X
Actionable	X	–	X	X	–	–	X	X	X	–	-
Theme 3: Infrastructure											
Support	–	X	X	X	X	X	X	X	X	–	X
Data Culture	–	–	–	–	X	–	X	X	–	–	X
Theme 4: Data Quality											
Data Accuracy	X	–	X	X	X	X	X	X	X	X	X
Data Validity	X	–	X	X	X	X	X	–	X	X	X
Theme 5: Risks											
Affective	X	–	X	–	X	X	X	–	X	–	X
Behavioural	X	–	–	X	X	X	X	–	X	X	X

Noting that these themes were generated by synthesising the results of all articles included within this review, they may not have been outlined as “themes” within their respective articles alone

We also present how the results have implications for both sensemaking and implementation in Table 6. Such table is grouped by the *“constituents of the sensemaking perspective”* [34], and further detail is outlined in the discussion.

#### Theme 1: data communication

Data communication encompassed how the data was both presented and interpreted.

*Presentation* Data presentation was a focal point to a number of comments. These included comments on data granularity [44, 45, 55, 63], frequency [44], complexity [63], and graphical representation [58].

There were some discrepancies in the preferred level of data granularity. Some respondents favoured individual-level data [45, 55] because it allowed them to focus on specific patient outcomes [55]. Other respondents outlined a preference for summative [63] or longitudinal data, as this allowed them to see trends [44]. Sometimes, respondents requested both forms of data with the ability to further explore when required [44]. Another suggestion was to just focus on high-risk patients alone [44].

There was commentary surrounding the presentation complexity. This was for a few reasons: too much information [63], relevance [63], or because data lacked sufficient description [58]. Respondents stressed that the presented data needed to be relevant [63], not complicated [63], and support was needed to ensure this [57].

*Interpretation* Interpretation was raised in two ways. First, how physicians and surgeons would interpret their own data [45, 56–58, 60, 63]. Second, how others would interpret data [60], in particular those who were outside of the clinical speciality [45, 62, 63]. Put succinctly, the context of this data was deemed important.

Respondents went through a process of interpretation when presented with data [56]. Interpretation captured a few different processes. For example, respondents sought clarification by either asking questions to, or requesting a facilitator was present [56, 57, 63]. Others contextualised the data by providing explanations or detail on the circumstances of an event [56].

Some respondents themselves reported difficulties interpreting the data [45, 57, 63]. In other cases, it was highlighted by some respondents that people outside the speciality may not correctly interpret data. This included non-clinical data handlers [62], the public [63], and hospital management [45]. Whilst those outside to the clinical speciality may have been seen to misinterpret the data, those within the speciality may be helpful in facilitating interpretation [55, 57].

#### Theme 2: performance reflection

Performance reflection captured how the physician or surgeon used data to reflect upon their existing practice and how this subsequently influenced their future practice.

*Attribution* Attribution captured how much the physician or surgeon believed they had influenced the data. Whether, upon reflection, they *attributed* data to their own personal actions, or factors outside of their control [45, 57, 60, 61, 63]. Many felt that data actually reflected the latter. External factors outside of the individual’s control were attributed to having caused some unfavorable reporting [44, 45, 55].

External factors included patient [55–57, 60, 63], system/reporting process [45, 56, 58, 61, 63], or financial factors [45]. Respondents highlighted that they felt their performance was being judged unfairly because of these external factors [44, 45]. For some, this was enough to disregard the data entirely and thus made no adjustments to their practice [45].

Despite the presence of external factors, some respondents recognised the outcome was still their responsibility [63].

*Actionable* Actionable captured how effective the data communicated what needed to be changed and how the data could be translated into future practice. Respondents who accepted the data reported they would take action to improve their practice [45, 57, 60, 61].

In some cases, respondents preferred having performance recommendations highlighted. For some, this included identification of specific areas to improve [57, 58, 60], highlighting high and low performing areas [58], and information from others as to how they improved their practice [58].

The format of information was also discussed in relation to the actionability of data [55]. Different formats were seen to offer different actionable insights. Longitudinal data allowed respondents to see trends, whilst granular data allowed a more focused approach towards patient outcomes [55]. Group data [55, 58] and peer comparisons [60, 61] was also a valuable motivator to drive practice change.

For reporting to be actionable for some, it needed to recommend relevant skill enhancement interventions [58]. This would allow the data to be translated into practice. Interventions included links to resources or clinical rationale [58].

#### Theme 3: infrastructure

Infrastructure captured the importance of support and culture when sharing data associated with performance.

*Support* Many requested a need for support alongside these data initiatives. Some reported a general need for resources [58], whilst others specifically expressed a need for additional literature [59], training/coaching [60], peer support [44, 56, 57, 60, 61, 63], and technology [59, 63].

In some cases, support was needed in conjunction with the data. This support was needed for two reasons. First, to support understanding, and second, to ensure the data led to practice change. Examples of such support included: providing literature on evidence-based measures [59], the presence of a facilitator in order to answers any questions [56], and the presence of a colleague to aid interpretation [57].

Support was also needed after the data had been presented. In order to improve practice, the data needed not to be viewed in isolation and thus support needed to reflect this. Respondents discussed closing the loop by revisiting data and prior recommendations to assess impact [59]. Others referenced consulting colleagues [63] and coaching [60].

Support was also needed indirectly as some reported competing priorities. Factors such as insufficient time, staffing, and other additional responsibilities were highlighted as barriers to such initiatives [44, 57]. Support would be needed to address these factors. This support would allow the data to be focused on and not create an additional burden [44, 45].

*Data Culture* Respondents also referenced the culture surrounding data sharing. When data was discussed openly and in a non-threatening way, group discussions were seen as helpful in driving performance improvement. This was for two reasons. First, respondents felt they could combine experiences and discuss ways to improve [63]. Second, group discussions were seen as catalysts for practice change [59]. There was also reference to systemic support and leadership that fostered a growth and learning culture [60]. This, coupled with a culture that promotes improvement lead to more data engaged practitioners [60]. In addition, some made reference to specifically using the data to learn and educate others who were less experienced [61].

#### Theme 4: data quality

Data quality captured physician and surgeon concerns surrounding data accuracy and validity.

*Data Accuracy* Concerns surrounding data accuracy were raised [44, 57–60, 62, 63]. In some cases this was in relation to data entry and assembly.

First, accuracy concerns stemmed from erroneous data entry. This was from either coding mistakes [58], or because those entering the data were not clinically trained [63].

Second, accuracy was also questioned in relation to data assembly. This was for a few reasons. Some felt that a single data source could not accurately measure performance. This was because some diseases, for example, were not captured in the data source [44] or because small samples did not provide an accurate picture of performance [45]. One respondent estimated only 10 – 20% of practice was accurately being presented [55]. Others had accuracy concerns when data source or collection processes were not transparent [62]; however, if deemed unbiased then the data was trusted [61].

*Data Validity* Data validity captured the level to which the data measured clinical performance. It was felt that the data were too simplistic and unable to represent the complexities within clinical practice [57, 62].

Data validity was also raised in relation to inappropriate comparisons [60, 63], data source [44, 55], and sample size [45, 58, 63].

First, inappropriate comparisons impacted data validity. Respondents believed they should not be compared to peers who practice differently [60, 63]. There were some solutions offered to improve this, which included stratifying samples [58] and ensuring that most “important” indicators were provided [57]. This process would allow for like-for-like valid comparisons as stressed in [56].

Second, data source also impacted data validity [55, 60], with some viewing certain data sources to be more valid than others. For example, respondents viewed the electronic health record as being more representative than other, more targeted, speciality specific systems [55]. In other cases, patient satisfaction and evaluations were not seen as valid representations of care [60].

Third, sample size impacted data validity [45, 58, 59, 62, 63] as respondents did not feel the data reflected their entire practice. In addition, larger sample sizes were needed to generate meaningful comparisons [59]. Respondents felt small sample sizes were misleading, particularly as the data could be taken out of context [45].

When respondents felt data was not a valid measure of their performance, they questioned the ability to use this as a generalised measure of performance [55].

#### Theme 5: risks

Risks, as a theme, captured how using data to promote performance improvement could have negative repercussions, be that affective or behavioural.

*Affective Risks* The sub-theme affective risks captured the negative affective expressed by physicians and surgeons when presented with clinical performance data. These emotional responses included anxiety [57], fear [59], guilt [44], helplessness [60], surprise/shock [55], and frustration [45].

Fear stemmed from the possibility of data usages beyond quality improvement and learning. Without contextualisation, respondents feared repercussions were an inevitability. These include punitive action [59] and a reduction in patient referrals [63]. In some cases, respondents felt threatened by data [57].

Expressions of guilt followed when data identified areas of improvement. This could be for a few reasons. For example, the data challenged perceptions of being high-performing [60], or because whilst most strive for the best patient outcomes [63], the data may imply that this may not be the case.

Other negative emotions, such as irritation and frustration were reported [45]. These emotions were expressed when respondents were not happy with their reporting. What is more, despite the performance perhaps requiring adjustment, these emotions were considered a barrier to changing subsequent behaviour [45].

*Behavioural Risks* The sub-theme behavioural risks captured the negative impact that data can have on physician and surgeon behaviour. The behavioural responses included cherry-picking low-risk patients [63], attempting to “fix” the reporting and not the practice [62, 63], discrediting the data as a bureaucratic exercise [58], and ignoring recommendations [44, 45, 55, 60].

Some respondents were aware of physicians and surgeons who had altered their patient case-mix in order to improve their outcome reporting [59, 63]. Patients considered high-risk were potentially denied treatments to improve reporting [63]. Thus, data reporting could have deleterious downstream effects on a patient through the denial of treatment.

Respondents discussed the risk of individuals who attempted to alter the data instead of their performance [60, 63]. Gaming behaviour was also reported [62]. In both instances, the behaviour change was not with the view to improve the quality of care or patient outcome, but to change how they are portrayed in the reporting.

Physicians and surgeons also reported making no behaviour changes after data reporting. This occurred when respondents felt the data did not represent their care [55, 63].

## Discussion

This review thematically synthesised literature where electronic health data initiated a review of clinical performance. Five themes (*data communication, performance reflection, infrastructure, data quality,* and *risks*) emerged from the analysis. In addition to the themes, the results also presented some additional observations that may have implications for sensemaking and/or practitioner CPD namely, the international context of the studies and the data dissemination processes.

In order to address the objective, the findings are discussed in conjunction with the existing literature surrounding sensemaking. We scaffold such discussion using the *“constituents of the sensemaking perspective”* presented by Sandberg and Tsoukas [34] (see Fig. 1), and discuss how the findings complement such perspective within this context. This includes, events that trigger sensemaking, the process of sensemaking, outcomes of sensemaking, and factors that influence sensemaking.

A succinct summary of how the findings fit within such perspective is presented in Table 6. Whilst only an initial contribution towards understanding sensemaking in this context, we present some important considerations that are specifically tailored to this context, and some implications for effective performance reflection, learning, and development are presented throughout. Further work is required to continue to build on such framework.

**Table 6:** A table to present how each of the findings are discussed within the key constituents of sensemaking [34] for this context. Such format and breakdown was adapted for this context from Sandberg and Tsoukas [34]

Constituents of sensemaking in the context of presenting performance data in healthcare.			
Events that trigger sensemaking	Process of sensemaking	Factors that influence sensemaking	Sensemaking outcomes
Presenting data could be considered a *planned event* that triggers the sensemaking process. Data quality issues may be considered an *unplanned event* that distract practitioners from the planned sensemaking processes. If data is deemed inaccurate as a result of the above process, data is disregarded and unlikely to lead to development. Focus should be placed on using the data for learning and development with acknowledgement that data is a tool to stimulate such processes. This could encourage the planned sensemaking process that leads to performance reflection, rather than a only focusing on data inaccuracies.	After sensemaking is triggered, cues are extracted from the data (creation process), interpreted, and decisions are made related to action. The many different preferences that exist related to data presentation highlight that practitioners extract different cues from a scenario, and what is meaningful to one practitioner may be different to the next. Data should be presented in a way that promotes exploration and discovery as this promotes a greater amount of sensemaking that has a greater affiliation with learning. Narratives can support sensemaking by providing appropriate context and scaffolding and interpretation can involve triangulating data from multiple data sources in order to create sense. Decisions about attribution are made during the sensemaking process, and data must be attributable to performance in order to be considered actionable. Focus must be placed on the learning and development purpose of such data to ensure that this aligns with what the practitioners see as the purpose.	Many factors influence sensemaking and include emotions, the presence of support, and collaborative cultures. Common emotional responses (e.g. fear, guilt and anxiety), may inhibit a practitioners ability to generate effective meaning. Cultures that promote collegial environments that encourage the use of data for such as purpose are seen to facilitate effective sensemaking. Support can also facilitate effective sensemaking and can include the presence of a facilitator, peers, or additional resources. International context is important consideration when reviewing data associated with performance due to different regulation and processes that may influence sensemaking.	Sensemaking stops when sense is restored and however a greater degree of sensemaking is needed in order to lead to learning. There is a risk that sensemaking leads to unfavorable behavioural change which highlights the needs to facilitate effective sensemaking by considering all of the ideas presented throughout this review (e.g. culture, context, and support) The above must be kept in mind to ensure that sensemaking leads to learning, development, and performance improvement.

### Events that trigger sensemaking

In the context of the findings, presenting data associated with performance could be considered a “planned event” that triggers sensemaking (see Fig. 1). It is considered planned because it was purposefully presented to the practitioner to support their development. Subsequently, the sensemaking process is ideally triggered to understand what is being presented, how it is related to clinical practice, and how such information can be used in order to inform future clinical practice. This is the desired intention; sensemaking being triggered to assign meaning to the data that leads to learning and development, which improves future practice.

The results of this review, however, present situations where the quality of such data was questioned. If data was considered inaccurate or invalid there seemed less corroboration with practice change, or the data is simply disregarded. This is highlighted in the *data quality* theme. In this case, the sensemaking process could have been triggered by an “*un*planned event” [34]; the data inaccuracies. Whilst the data was presented in a planned manner, the sensemaking efforts have instead shifted to focus on such deficiencies. This was not planned. Given that the sensemaking efforts are instead focused on finding and understanding data inaccuracies, this has potentially distracted from the main aim of presenting such data. As a result, the data is deemed unfit for purpose. Such a situation is also emphasised by Weick [30], who highlighted that sensemaking triggers are a result of an individuals own making. They are a result of certain aspects of a scenario being, or not being, attended to. In this case, the sensemaking efforts have been triggered by attending to the data quality concerns (unplanned), which has moved the practitioner away from the performance reflection/development activity (planned). Thus, data inaccuracies do not just lead to distrust in data, but also distract from the planned, and more beneficial sensemaking process mentioned earlier.

Similar data quality issues are also highlighted to impact sensemaking in more traditional educational settings; when data is deemed inaccurate, less attention was paid to it [38]. An important observation as only data that is considered accurate and “salient” had connections with planned behaviour change associated with learning [38]. Put simply, data that is disregarded cannot lead to learning, development, or practice change. Whilst seemingly obvious, this is an important consideration for policy, professional frameworks, and regulatory guidelines. This is because such activities are being recorded as CPD activities, and therefore must have implications for development; rather than become a “tick-box” exercise to fulfil a requirement to clinically practice (alike that reported by Macdougall, Epstein and Highet [64]). Further work is required to assess how this can be both accurately monitored and integrated with CPD point/credit systems [4].

Whilst we agree that data quality issues should be minimised, and the that highest quality data should be presented, we recognise that this is challenging. Big data in healthcare is notoriously complex, and this has created significant challenges for access, processing, and analysis [65]. To account for the data quality issues raised within the findings, and also the aforementioned data challenges, we argue for a change in the approach to data in this context. Focus, instead, needs to be placed on using this data to prompt performance reflection, and, as a result, learning and development. Data is just one tool that can initiate such processes; it is not to be used punitively or, in this case, for clinical diagnosis, for example. By being open and up front about such an approach, practitioners may accept that the data may not be “perfect”, but that it is indeed providing insights or thought provoking prompts about performance that otherwise may be invisible. Taking such an approach increases the likelihood of effective sensemaking (based on the planned development activity), which leads to performance reflection, development, and, ultimately, performance improvement, whilst also removing the focus on data inaccuracies or shortfalls. However, another point to highlight, is that data quality issues directly from erroneous data entry by practitioners [66, 67], may improve if practitioners are given the opportunity and resources to reflect upon such data regularly. In short, the process of reflecting on data may improve its quality cyclically, making it more effective for future reflection. This not only has benefits for the practitioners future development activities; but also potentially the overall quality of care more broadly, as erroneous data entry could have significant ramifications.

### The process of sensemaking

After sensemaking is triggered, “making sense” occurs through cyclic processes of creation, interpretation, and enactment in an attempt to restore sense [34] (see Fig. 1). The themes *data communication* and *performance reflection* found within this review provide some insights into such processes in this context.

*Creation* is when key elements of information are extracted from a scenario, which then go on to facilitate interpretation [30, 34] (see Fig. 1). The lack of consensus in the findings surrounding *data presentation* preferences, not only highlights the difficulty in recommending one approach to presenting data, but also emphasises how individualistic the sensemaking process is. Here, it is exemplified within the creation process. Practitioners attended to and extracted different cues from data, inferring that what is deemed meaningful for one person, may not be meaningful for all. Given this challenge, instead of focusing on the visual specificities (e.g. types of graph or data granularity), we shift our focus to how data can be presented to facilitate sensemaking that, given the context, leads to learning. To understand this, we draw on Marchionini [68] who differentiated between retrieving and seeking information. Retrieving involves simply finding and extracting pre-existing information (e.g. reviewing a statistic). Seeking requires more effort than retrieval as the information may not currently exist. A practitioner may have to spend more time exploring and triangulating elements of the data in order to draw insights. This process requires a greater degree of sensemaking, which Marchionini [68] linked to a greater degree of learning. Therefore, data that is presented in a way that promotes a greater exploration and discovery could be more beneficial for practitioner learning and development.

Also relevant to the data presentation theme found within this review, is the inclusion of narratives to complement data to support the creation process. These can help shape meaning that is effective for learning and development. Narratives may aid the practitioner in contextualising the data; thus, allowing them to extract cues that are appropriate and relevant for interpretation. Chalil Madathil and Greenstein [69] found that narratives increased personal relevance and subsequent data meaning. This allows the individual to relate to the measures and visualise themselves within the depicted scenarios [69] during the enactment process. Thus, increasing their levels of data engagement. The results reported here were consistent with such reporting, both in highlighting the importance of data communication and personal preferences . Each of which needs consideration when developing guidelines and policy for data informed performance reflection in healthcare.

*Interpretation and enactment* is when the cues that were extracted during the creation process are elaborated on to develop a more detailed account of a scenario, and then based on this, action is taken [34] (see Fig. 1). Action can be taken through internal enactments/simulation, or through physical activity [34]. Given that these ideas are often intertwined within the literature, likely because they are intertwined in reality, we discuss these ideas together in conjunction with the findings.

First, *interpretation*, was found as sub-theme within this review. Raj, Lee, Garrity and Newman [70] proposed that when engaging with health data specifically, the sensemaking process involves a series of analytical interpretation activities. These included: overlaying context specific trends, triangulating information from distinct data points, internally simulating scenarios, and hypothesising alternative outcomes. They argued that their work supports the work of Klein, Phillips, Rall and Peluso [71], and made recommendations for designing tools to support data sensemaking. These included ensuring data self-validation through triangulation across multiple data indicators, presenting the temporal nature of data (i.e. trends over time), and the inclusion of future prediction, to account for internal simulation of scenarios.

The findings of this review also highlight that when interpreting data, practitioners also make decisions about how attributable the data was to their performance. This was highlighted in the *performance reflection* theme, which encompassed both data *attribution*, and how *actionable* the data was deemed. This has important implications given that attribution and action are already linked within sensemaking literature [72]. Data has been considered actionable by users if they both trusted the data curation process and considered the data fit for purpose [73]. These internal decisions impacted whether the end-user took action as a result [72, 73]. This links back to the ideas introduced earlier surrounding data quality; emphasis needs to be placed on the learning and development purpose of such data. It may be correct that the data would not be fit for the purpose of public reporting or dissemination, for example, but it may fit for purpose to help initiate thought provoking insights on personal performance. These are two very different purposes and require a significant shift in culture and approach to data. However, if this shift is made, the data may be considered “fit for purpose” and therefore useful for action.

### Factors that influence sensemaking

Sensemaking efforts do not take place in isolation, instead they are shaped by the factors and context in which they take place [34] (see Fig. 1). The list of such factors is “almost endless” [34], but some of the more prevalent factors within the literature were also reported within the findings. These include emotion *(affective risks)*, *support*, and *culture* that were highlighted as sub-themes, and also the context highlighted by characteristics of the included studies.

Emotion is widely reported to impact how an individual makes sense of a situation [29, 36, 74]. Generally, negative emotions inhibit sensemaking [75], whilst positive emotions facilitate sensemaking [76]. Given the negative emotions reported in the findings (e.g. anxiety, guilt and fear), we focus on their impact. Such emotions have been shown to hamper sensemaking [34]. This is because they require cognitive processing that takes away from the processing required to effectively notice and extract crucial information from a scenario which is required for effective sensemaking [34, 77, 78]. Given the heightened emotional response surrounding performance reporting/data, and feedback more generally, it is important to highlight that this could be impacting how an individual is making sense of a situation. Specifically, that a practitioner may not be able to generate actionable information from the data, as result of their emotional state. Such affective behaviours have also been reported when leveraging data within national healthcare policy [79]. It has therefore, been recommended that a more tailored approach is taken when handling data associated with performance [79] and policy should reflect this. Namely, ensuring that practitioners feel safe and comfortable reviewing such data through placing emphasis on using it to facilitate learning and development, not for other activities (e.g. public reporting or performance management).

Support was highlighted within the results and was seen to scaffold data. When effective, scaffolding has been shown to facilitate sensemaking and is argued to be essential for learning [80]. Group support also benefits professional development [81], leads to sustained learning [82, 83], and strengthens health systems [83]. Thus, highlighting a need for collegial discussions and a collaborative culture in order to successfully implement healthcare improvement initiatives [84]. This is further supported by regulatory bodies internationally [8, 12, 14].

Culture is also shown to impact how an individual makes sense of a scenario [29, 68, 71, 72]. *“[S]ensemaking never takes place in isolation but always in specific contexts”* (p.S15) [34] and within their review Sandberg and Tsoukas [34] found that 46% of included studies noted the impact of context on sensemaking. Particularly relevant to this work, is the impact of “social” and “institutional” contexts. Society tied individuals to decisions that were constrained by social norms and expectations, it influenced the salience of information, and, as a result, provided boundaries for justifiable actions. Practitioners are therefore likely to (re)act, based on a function of their surroundings. Therefore, a culture that promotes using data for clinical performance improvement, development, and learning is more likely to lead to effective sensemaking that generates development and improvement. Health institutions must *“... embrace the value of data to drive improved outcomes of care”* (p.125) [2] and promote a non punitive environment to facilitate discussions around success and failure as depicted by data [2]. This is consistent with other work surrounding healthcare digitisation [85] that recommends healthcare organisations promote a strong data culture in order for digital technologies to impact behaviour.

The professional performance framework within Australia [8] promotes a culture that fosters a commitment from practitioners to engage in reflective practice, lifelong learning, and collegial support. Encouraging both individual *and* shared knowledge generation, alongside encouraging practice transformation, is important for continuing professional development [86]. Cultures that fostered such dynamics were associated with more adaptive behaviours that allowed individuals to adjust to new ways of learning through technology, performance development initiatives, and inter-professional discussions [86]. These strong team dynamics also lead to more positive emotions, deeper levels of sensemaking, and greater group agreement [87]. This compliments the many reported benefits of group meetings and knowledge sharing healthcare [88, 89], and signals their importance for group data sensemaking. Thus, group meetings to discuss and reflect on clinical performance data should be encouraged.

Having discussed the impact that emotion, support, and culture have on sensemaking in this context , we shift the focus to the impact of context. All studies included within this review were from North America (Canada & USA). This is important to highlight given countries have different regulatory guidelines surrounding activities such as CPD, and also how CPD links to other requirements such as registration. As presented within the background section, whilst countries such as Australia, UK, and Canada require practitioners to demonstrate development activities in order to practice [7–9], the exact requirements differ. For example, in Australia from January 2023, it is a *new* requirement for practitioners to spend a stipulated amount of time actively reviewing their performance data [8]. The requirements also vary within countries across professions [9]. In order to account for international differences, and fully integrate data informed learning and development in healthcare, further work needs to be done to ensure the processes account and complement international development frameworks, clinical governance, and accreditation standards. This is within scope of the research area practice analytics [4].

All of the contextual factors above have implications for sensemaking. This is particularly the case given the variety of different international expectations and processes highlighted above, and also the different data dissemination processes highlighted in the results. To illustrate such point, we highlight three examples. First, practitioners who have experience publicly releasing performance data may approach data differently to a practitioner who does not have such experience. A second example is whether reviewing performance data is a compulsory activity or not, as there may be different underlying motivations at play. A final example are differences across public and private systems, to which their may be contrasting priorities/expectations. These situational factors may impact what data is extracted within the creation process, how it is interpreted, and how it is acted upon (the sensemaking process). This is in conjunction with different levels of emotion and experiences that may mediate the whole process. Taken together, there must be both strong emphasis placed on using this data for learning and development, in an attempt to mitigate any predetermined biases, but also recognition that sensemaking is inevitably highly individual. What is meaningful in one case, or for one practitioner, may be different to the next. Hence, we argue for more routine access to performance data that allows practitioners to self-regulate and explore their own performance and development needs based on their *own* sensemaking.

Also related to context, *mis*interpretation of data was also highlighted if the data was taken out of context. Concerns were raised about others, outside of their practice, incorrectly interpreting practitioner performance data. This has parallels to the ideas presented earlier about international context. Individuals extract cues from a situation based on what they deem meaningful, thus, what a practitioner reviews about their individual practice may be different to an external person reviewing the data. The latter is less likely to have the same amount of details (context) surrounding the data in order to interpret the information in the same way.

### Outcomes of sensemaking

The ultimate outcome of sensemaking is that sense is restored, and at that point, sensemaking stops [34] (see Fig. 1). However, it is acknowledged that only a “plausible” account of a scenario is needed to stop sensemaking, not necessarily an accurate one [30, 34]. This means that if data is presented, and the cues extracted during the creation process lead to some form of restoration in sense, then sensemaking will cease. The results highlight occasions where this was potentially the case, and instead behaviour changed unfavourably (*behavioural risks*). This reinforces the significance of appropriate data presentation that facilitates a greater amount of sensemaking (through exploration and discovery), cultures and contexts, and strong levels of support. Important, given that the ultimate goal of presenting data to practitioners, in this case, is that it leads to learning, development, and improvement.

All of the above emphasises the pivotal role that sensemaking plays in this context; it supports the transformation of data to learning and development. As such, highlights the significance of both, the ideas presented throughout this discussion, and that further work is needed to explore sensemaking in this context.

## Conclusion

This review is the first attempt to explore data sensemaking in the context of practice analytics. It outlines some prevalent themes associated with using data to reflect on clinical performance. When these themes are reviewed in conjunction with existing sensemaking and healthcare research they point to some important areas for consideration. For one, there are many factors that could be impacting how an individual is “making sense” of their data inclusive of context, emotion, culture, and levels of support. Not only could the process itself be impacted by such factors, but this can have ramifications on future behaviour.

This review emphasises a clear gap. No research has specifically explored how medical practitioners make sense of electronic health data associated with their clinical performance. This may be because it is a difficult phenomenon to observe and measure, with very few instruments or tools to do this. Whilst this review attempted to explore such phenomenon, the review relied solely on secondary analysis of research that qualitatively explored more general experiences with such data, and reviewed the findings with existing sensemaking literature. No research found or included explored data *sensemaking* specifically. Further work must explore this process and also factors that that may affect this. This is a clear research stream and objective within practice analytics [4]. In doing so, further recommendations for policy and guidelines can be made to ensure that data is both meaningful and positively impacts future practice.

### Limitation of evidence

This review is not without its limitations. First, the review is only inclusive of articles that are captured by the search strategy. Whilst the researchers endeavoured to be exhaustive, if articles used different terminology, MeSH terms, or were not indexed in the databases searched, they would not have been found.

Second, the review uses secondary analysis to address its research objective. This involved the inclusion of papers that were not exploring the sensemaking process. Further research that specifically aims to explore this process is required and necessary in order to further understand the sensemaking process that is enacted when physicians and surgeons engage with data associated with their performance.

## Supplementary Information

**Additional file 1.** Supplementary Material: Appendix.

## Data Availability

All data generated or analysed during this study are included in this published article. All articles included in this systematic review are available using the references included in the reference section of this review.

## Abbreviations

CERQual: Confidence in the evidence from reviews of qualitative research
CPD: Continued professional development
MeSH: Medical subject headings
PRISMA: Preferred reporting items for systematic reviews and meta-analyses guidelines
SPIDER: Sample, phenomenon of interest, design, evaluation, research type
SRQR: Standards for reporting qualitative research
