# Supplementary material for: An exploration into physician and surgeon data sensemaking: a qualitative systematic review using thematic synthesis
Source: BMC Med Inform Decis Mak. 2022 Sep 28;22:256. doi: 10.1186/s12911-022-01997-1 (PMC9520820; doi:10.1186/s12911-022-01997-1)
Supplement: Supplementary file 1 — Additional file 1. Supplementary Material: Appendix. [file 12911_2022_1997_MOESM1_ESM.pdf]

Appendix

Table A1 Extended MEDLINE line by line search strategy.

Extended MEDLINE Search Terms		
SPIDER	Line	Search Term
Sample	1	Exp Physicians/
	2	Physicians, Family/
	3	Physicians, Primary Care/
	4	Physicians, Women/
	5	Cardiologists/
	6	Pharmacology, Clinical/
	7	Endocrinologist/
	8	Gastroenterologist/
	9	Geriatricians/
	10	Allergists/
	11	Oncologists/
	12	Nephrologists/
	13	Neurologists/
	14	Rheumatologist/
	15	Pediatricians/
	16	Neonatologists/
	17	Pulmonologists/
	18	Pathologists/
	19	Dermatologists/
	20	Surgeons/
	21	Urologists/
	22	Neurosurgeons/
	23	Orthopedic Surgeons//
	24	Otolaryngologists/
	25	Physician*.mp.
	26	Cardiologist*.mp.
	27	Geneticist*.mp.
	28	Pharmacologist*.mp.
	29	Endocrinologist*.mp.
	30	Gastroenterologist*.mp.
	31	Geriatrician*.mp.
	32	Haematologist*.mp.
	33	Hepatologist*.mp.
	34	Immunologist*.mp.
	35	Allergist*.mp.
	36	Oncologist*.mp.
	37	Nephrologist*.mp.
	38	Neurologist*.mp.
	39	Rheumatologist*.mp.
	40	Paediatrician*.mp.
	41	Neonatologist*.mp.
	42	Pulmonologist*.mp.
	43	Pathologist*.mp.
	44	Dermatologist*.mp.
	45	Surgeon*.mp.
	46	Urologist*.mp.
	47	Neurosurgeon*.mp.
	48	Otolaryngologist*.mp.
	49	or/1-48
Phenomenon of Interest	50	Feedback/
	51	Feedback, Psychological/
	52	Formative Feedback/
	53	'Knowledge of Results, Psychological'/
	54	Benchmarking/
	55	Employee Performance Appraisal/
	56	Performance ADJ2 (Clinician* or Doctor* or Physician* or Cardiologist* or Geneticist* or Pharmacologist* or Endocrinologist* or Gastroenterologist* or Geriatrician* or Haematologist* or Hepatologist* or Immunologist* or Allergist* or Oncologist* or Nephrologist* or Neurologist* or Rheumatologist* or Paediatrician* or Neonatologist* or Pulmonologist* or Pathologist* or Dermatologist* or Surgeon* or Urologist* or Neurosurgeon* or Otolaryngologist*).mp.
	57	Performance ADJ2 (Evaluat* or Measur* or Report* or Scorecard* or Indicat* or Review* Rat*).mp.

Continuation of Extended MEDLINE Search		
SPIDER	Line	Search Term
	58	Performance ADJ3 (Reflect* or Apprais* or Information).mp.
	59	Performance Data.mp.
	60	Performance Metric*.mp.
	61	Personal* Performance.mp.
	62	Individual* Performance.mp.
	63	Clinical Performance.mp.
	64	Medical Performance.mp.
	65	Professional Performance.mp.
	66	Performance Dashboard*.mp.
	67	Practice Performance.mp.
	68	Feedback.mp.
	69	Feed Back.mp.
	70	Continu* Professional Development.mp.
	71	Life Long Learning.mp.
	72	Benchmark*.mp.
	73	(Refle* ADJ2 Practice).mp.
	74	(Colleague* or Peer) ADJ2 (Compar* or Average* or Relation* or Performance).mp.
	75	or/50-74
Research Design	76	Focus Groups/
	77	Grounded Theory/
	78	Interview/
	79	Interviews as Topic/
	80	Qualitative Research/
	81	Focus Group*.mp.
	82	Qualitativ*.mp.
	83	Interview*.mp.
	84	Mix* Method*.mp.
	85	Quasi*.mp.
	86	Grounded Theory.mp.
	87	Theme*.mp.
	88	Thematic*.mp.
	89	Open-ended.mp.
	90	or/76-89
Summary	91	49 AND 75 AND 90
	92	Limit 91 to yr="2010 - 2021"
	93	Limit 92 to English Language
End of Extended MEDLINE Search		

Table A2 EMBASE line by line search strategy.

EMBASE Search Terms		
SPIDER	Line	Search Term
Sample	1	Exp Physicians/
	2	Emergency Physician/
	3	Female Physician/
	4	Foreign Physician/
	5	Hospital Physician/
	6	Cardiologist/
	7	Clinical Pharmacology/
	8	Endocrinologist/
	9	Gastroenterologist/
	10	Geriatrician/
	11	Exp Oncologist/
	12	Nephrologists/
	13	Neurologists/
	14	Rheumatologist/
	15	Exp Paediatricians/
	16	Neonatologists/
	17	Pulmonologists/
	18	Pathologists/
	19	Exp Dermatologists/

Continuation of EMBASE Search		
SPIDER	Line	Search Term
	20	Exp Surgeon/
	21	Exp Urologist/
	22	Otolaryngologists/
	23	Infectious Disease Specialist/
	24	Exp Haematologist/
	25	Hepatologist/
	26	Physician*.mp.
	27	Cardiologist*.mp.
	28	Geneticist*.mp.
	29	Pharmacologist*.mp.
	30	Endocrinologist*.mp.
	31	Gastroenterologist*.mp.
	32	Geriatrician*.mp.
	33	Haematologist*.mp.
	34	Hepatologist*.mp.
	35	Immunologist*.mp.
	36	Allergist*.mp.
	37	Oncologist*.mp.
	38	Nephrologist*.mp.
	39	Neurologist*.mp.
	40	Rheumatologist*.mp.
	41	Paediatrician*.mp.
	42	Neonatologist*.mp.
	43	Pulmonologist*.mp.
	44	Pathologist*.mp.
	45	Dermatologist*.mp.
	46	Surgeon*.mp.
	47	Urologist*.mp.
	48	Neurosurgeon*.mp.
	49	Otolaryngologist*.mp.
	50	or/1-49
Phenomenon of Interest	51	Benchmarking/
	52	Constructive Feedback/
	53	Feedback System/
	54	Negative Feedback/
	55	Positive Feedback/
	56	Psychological Feedback/
	57	Performance ADJ2 (Clinician* or Doctor* or Physician* or Cardiologist* or Geneticist* or Pharmacologist* or Endocrinologist* or Gastroenterologist* or Geriatrician* or Haematologist* or Hepatologist* or Immunologist* or Allergist* or Oncologist* or Nephrologist* or Neurologist* or Rheumatologist* or Paediatrician* or Neonatologist* or Pulmonologist* or Pathologist* or Dermatologist* or Surgeon* or Urologist* or Neurosurgeon* or Otolaryngologist*).mp.
	58	Performance ADJ2 (Evaluat* or Measur* or Report* or Scorecard* or Indicat* or Review* Rat*).mp.
	59	Performance ADJ3 (Reflect* or Apprais* or Information).mp.
	60	Performance Data.mp.
	61	Performance Metric*.mp.
	62	Personal* Performance.mp.
	63	Individual* Performance.mp.
	64	Clinical Performance.mp.
	65	Medical Performance.mp.
	66	Professional Performance.mp.
	67	Performance Dashboard*.mp.
	68	Practice Performance.mp.
	69	Feedback.mp.
	70	Feed Back.mp.
	71	Continu* Professional Development.mp.
	72	Life Long Learning.mp.
	73	Benchmark*.mp.
	74	(Refle* ADJ2 Practice).mp.
	75	(Colleague* or Peer) ADJ2 (Compar* or Average* or Relation* or Performance).mp.
	76	or/51-75
Research Design	77	Exp Interview/
	78	Exp Qualitative Research/
	79	Qualitative Analysis/
	80	Focus Group*.mp.

Continuation of EMBASE Search		
SPIDER	Line	Search Term
	81	Qualitativ*.mp.
	82	Interview*.mp.
	83	Mix* Method*.mp.
	84	Quasi*.mp.
	85	Grounded Theory.mp.
	86	Theme*.mp.
	87	Thematic*.mp.
	88	Open-ended.mp.
	89	or/77-88
Summary	90	50 AND 76 AND 89
	91	Limit 90 to yr="2010 - 2021"
	92	Limit 91 to English Language
End of EMBASE Search		

Table A3 PsychInfo line by line search strategy.

PsychInfo Search Terms		
SPIDER	Line	Search Term
Sample	1	Exp Physicians/
	2	Physician*.mp.
	3	Cardiologist*.mp.
	4	Geneticist*.mp.
	5	Pharmacologist*.mp.
	6	Endocrinologist*.mp.
	7	Gastroenterologist*.mp.
	8	Geriatrician*.mp.
	9	Haematologist*.mp.
	10	Hepatologist*.mp.
	11	Immunologist*.mp.
	12	Allergist*.mp.
	13	Oncologist*.mp.
	14	Nephrologist*.mp.
	15	Neurologist*.mp.
	16	Rheumatologist*.mp.
	17	Paediatrician*.mp.
	18	Neonatologist*.mp.
	19	Pulmonologist*.mp.
	20	Pathologist*.mp.
	21	Dermatologist*.mp.
	22	Surgeon*.mp.
	23	Urologist*.mp.
	24	Neurosurgeon*.mp.
	25	Otolaryngologist*.mp.
	26	or/1-25
Phenomenon of Interest	27	Feedback/
	28	Knowledge of Results/
	29	Formative Assessment/
	30	Performance ADJ2 (Clinician* or Doctor* or Physician* or Cardiologist* or Geneticist* or Pharmacologist* or Endocrinologist* or Gastroenterologist* or Geriatrician* or Haematologist* or Hepatologist* or Immunologist* or Allergist* or Oncologist* or Nephrologist* or Neurologist* or Rheumatologist* or Paediatrician* or Neonatologist* or Pulmonologist* or Pathologist* or Dermatologist* or Surgeon* or Urologist* or Neurosurgeon* or Otolaryngologist*).mp.
	31	Performance ADJ2 (Evaluat* or Measur* or Report* or Scorecard* or Indicat* or Review* Rat*).mp.
	32	Performance ADJ3 (Reflect* or Apprais* or Information).mp.
	33	Performance Data.mp.
	34	Performance Metric*.mp.
	35	Personal* Performance.mp.
	36	Individual* Performance.mp.
	37	Clinical Performance.mp.
	38	Medical Performance.mp.

Continuation of PsychInfo Search		
SPIDER	Line	Search Term
	39	Professional Performance.mp.
	40	Performance Dashboard*.mp.
	41	Practice Performance.mp.
	42	Feedback.mp.
	43	Feed Back.mp.
	44	Continu* Professional Development.mp.
	45	Life Long Learning.mp.
	46	Benchmark*.mp.
	47	(Refle* ADJ2 Practice).mp.
	48	(Colleague* or Peer) ADJ2 (Compar* or Average* or Relation* or Performance).mp.
	49	or/27-48
Research Design	50	Exp Interviews/
	51	Exp Qualitative Methods/
	52	Focus Group*.mp.
	53	Qualitativ*.mp.
	54	Interview*.mp.
	55	Mix* Method*.mp.
	56	Quasi*.mp.
	57	Grounded Theory.mp.
	58	Theme*.mp.
	59	Thematic*.mp.
	60	Open-ended.mp.
	61	or/50-60
Summary	62	26 AND 49 AND 61
	63	Limit 62 to yr="2010 - 2021"
	64	Limit 63 to English Language
End of PscyhInfo Search		

Table A4 Cochrane Central Register of Controlled Trials line by line search strategy.

Cochrane Central Register of Controlled Trials Search Terms		
SPIDER	Line	Search Term
Sample	1	Exp Physicians/
	2	Physician Family/
	3	Physicians, Primary Care/
	4	Physicians, Women/
	5	Physician*.mp.
	6	Cardiologist*.mp.
	7	Geneticist*.mp.
	8	Pharmacologist*.mp.
	9	Endocrinologist*.mp.
	10	Gastroenterologist*.mp.
	11	Geriatrician*.mp.
	12	Haematologist*.mp.
	13	Hepatologist*.mp.
	14	Immunologist*.mp.
	15	Allergist*.mp.
	16	Oncologist*.mp.
	17	Nephrologist*.mp.
	18	Neurologist*.mp.
	19	Rheumatologist*.mp.
	20	Paediatrician*.mp.
	21	Neonatologist*.mp.
	22	Pulmonologist*.mp.
	23	Pathologist*.mp.
	24	Dermatologist*.mp.
	25	Surgeon*.mp.
	26	Urologist*.mp.
	27	Neurosurgeon*.mp.
	28	Otolaryngologist*.mp.
	29	or/1-28

Continuation of Cochrane Central Register of Controlled Trials Search		
SPIDER	Line	Search Term
Phenomenon of Interest	30	Feedback/
	31	Feedback, Psychological/
	32	Employee Performance Appraisal/
	33	Performance ADJ2 (Clinician* or Doctor* or Physician* or Cardiologist* or Geneticist* or Pharmacologist* or Endocrinologist* or Gastroenterologist* or Geriatrician* or Haematologist* or Hepatologist* or Immunologist* or Allergist* or Oncologist* or Nephrologist* or Neurologist* or Rheumatologist* or Paediatrician* or Neonatologist* or Pulmonologist* or Pathologist* or Dermatologist* or Surgeon* or Urologist* or Neurosurgeon* or Otolaryngologist*).mp.
	34	Performance ADJ2 (Evaluat* or Measur* or Report* or Scorecard* or Indicat* or Review* Rat*).mp.
	35	Performance ADJ3 (Reflect* or Apprais* or Information).mp.
	36	Performance Data.mp.
	37	Performance Metric*.mp.
	38	Personal* Performance.mp.
	39	Individual* Performance.mp.
	40	Clinical Performance.mp.
	41	Medical Performance.mp.
	42	Professional Performance.mp.
	43	Performance Dashboard*.mp.
	44	Practice Performance.mp.
	45	Feedback.mp.
	46	Feed Back.mp.
	47	Continu* Professional Development.mp.
	48	Life Long Learning.mp.
	49	Benchmark*.mp.
	50	(Refle* ADJ2 Practice).mp.
	51	(Colleague* or Peer) ADJ2 (Compar* or Average* or Relation* or Performance).mp.
	52	or/30-51
Research Design	53	Interview/
	54	Qualitative Research/
	55	Exp Interviews as Topic/
	56	Focus Group*.mp.
	57	Qualitativ*.mp.
	58	Interview*.mp.
	59	Mix* Method*.mp.
	60	Quasi*.mp.
	61	Grounded Theory.mp.
	62	Theme*.mp.
	63	Thematic*.mp.
	64	Open-ended.mp.
	65	or/53-64
Summary	66	29 AND 52 AND 65
	67	Limit 66 to yr="2010 - 2021"
	68	Limit 67 to English Language
End of Cochrane Central Register of Controlled Trials Search		

Table B1 Quality score for each paper using the SRQR. Y denotes that the requirement was met, and N denotes that the requirement was not met.

Article	Quality Score	S1	S2	S3	S4	S5	S6	S7	S8	S9	S10	S11	S12	S13	S14	S15	S16	S17	S18	S19	S20	S21
Barber et al. [55]	95.2%	N	Y	Y	Y	Y	Y	Y	Y	Y	Y	Y	Y	Y	Y	Y	Y	Y	Y	Y	Y	Y
Cooke et al. [56]	90.5%	N	Y	Y	Y	Y	Y	Y	Y	Y	Y	Y	Y	Y	Y	Y	Y	Y	Y	Y	Y	N
Desveaux et al. [57]	100.0%	Y	Y	Y	Y	Y	Y	Y	Y	Y	Y	Y	Y	Y	Y	Y	Y	Y	Y	Y	Y	Y
Eden et al. [58]	71.4%	N	Y	Y	Y	Y	N	N	N	Y	Y	Y	N	Y	Y	N	Y	Y	Y	Y	Y	Y
Ivanovic et al. [59]	71.4%	N	Y	Y	Y	Y	Y	Y	N	Y	Y	Y	Y	Y	Y	N	Y	N	Y	Y	N	N
Ivers et al. [44]	85.7%	N	Y	Y	Y	Y	N	Y	Y	Y	Y	Y	Y	Y	Y	Y	Y	Y	Y	Y	Y	N
Kamhawy, Chan and Mondoux [60]	95.2%	N	Y	Y	Y	Y	Y	Y	Y	Y	Y	Y	Y	Y	Y	Y	Y	Y	Y	Y	Y	Y
Laur et al. [61]	100.0%	Y	Y	Y	Y	Y	Y	Y	Y	Y	Y	Y	Y	Y	Y	Y	Y	Y	Y	Y	Y	Y
Payne and Hysong [45]	90.5%	N	Y	Y	Y	Y	N	Y	Y	Y	Y	Y	Y	Y	Y	Y	Y	Y	Y	Y	Y	Y
Szymczak, Feemster, Zaoutis and Gerber [62]	95.2%	N	Y	Y	Y	Y	Y	Y	Y	Y	Y	Y	Y	Y	Y	Y	Y	Y	Y	Y	Y	Y
Yi et al. [63]	95.2%	Y	Y	Y	Y	Y	N	Y	Y	Y	Y	Y	Y	Y	Y	Y	Y	Y	Y	Y	Y	Y

Table B2 A table to highlight the confidence in the findings at a sub-theme level based upon CERQual [50, 49].

Sub-theme	Articles that contributed to such finding	Methodological limitations	Coherence	Adequacy	Relevance	CERQual assessment of confidence in the evidence
			**Theme 1: Data Communication**			
Presentation	[55, 58, 44, 45, 63]	*Minor concerns:* three studies with minor concerns, two with moderate concerns. Concerns included lack of researcher reflexivity, sample size, and use of secondary analysis.	*Little to no concerns:* three studies with no concerns, and two with minor concerns. Concerns included lack of detail in free-text survey answers, and lack of elaboration in areas.	*Minor concerns:* two studies included less data.	*Moderate concerns:* all five studies considered moderate. Some did not focus on individual cognitive experiences and instead focused on other elements of the reporting or alternative aim e.g. model development.	*Moderate confidence*
Interpretation	[55, 56, 57, 44, 60, 45, 62, 63]	*Minor concerns:* three studies with no concerns, four studies with minor concerns, and one with moderate concerns. Concerns included social desirability of group settings, researcher reflexivity, and relevance of secondary analysis.	*Little to no concerns:* seven studies with no concerns, and one with minor. Concern was with one study than did not provide a lot of detail on finding.	*No concerns:* majority of studies offered a good level of data to support this finding.	*Moderate concerns:* two studies had minor concerns, five had moderate concerns, and one had high concerns. Concerns included very specific sample, context, and broader aims of the study.	*High confidence*
			**Theme 2: Performance Reflection**			
Attribution	[55, 57, 58, 60, 61, 45, 63]	*Minor concerns:* three studies with no concerns, two with minor concerns, and two with moderate concerns. Concerns included lack of researcher reflexivity, relevance of secondary analysis, and lack of detail surrounding sample sizes.	*Minor concerns:* six with no concerns, and one with moderate concerns. Concerns included on small/limited reference to finding.	*Minor concerns:* whilst covered in a large proportion of studies, the finding was not labelled in the same way throughout.	*Moderate concerns:* two studies with minor concerns and five with moderate concerns. Concerns included very specific samples and broad focus of studies.	*Moderate confidence*
Actionable	[55, 57, 58, 60, 61, 45]	*Minor concerns:* three studies with no concerns, one with minor concerns, and two with moderate concerns. Concerns included small and specific sample size, lack of alignment with questions and objectives due to secondary analysis, and lack of detail on sample characteristics.	*Little to no concerns:* four studies with no concerns, and two with minor concerns. Concerns included only small amounts of detail within data.	*Minor concerns:* coverage across the majority of articles but a variety of different terminology and descriptions used.	*Moderate concerns:* two studies with minor concerns, the remainder had moderate concerns due to lack of focus on internal processes/wider research aims.	*Moderate confidence*
			**Theme 3: Infrastructure**			
Support	[56, 57, 58, 59, 44, 60, 61, 45, 63]	*Minor concerns: three studies with no concerns, three with minor concerns, and three with moderate concerns. Concerns included lack of detail about researcher reflexivity, use of secondary analysis, and details about sampling decisions.*	*Minor concerns:* seven studies with no concerns, one with minor concerns, and one with moderate concerns. Concerns included only discussed briefly, and in one case, by one respondent.	*No concerns:* Covered in majority of studies and most had a lot of detail.	*Moderate concerns:* two studies minor concerns, remainder were moderate concerns due to lack of broader aims.	*Moderate confidence*

Table B3 A continuation of a table to highlight the confidence in our findings at a sub-theme level based upon CERQual [50, 49].

Sub-theme	Articles that contributed to such finding	Methodological limitations	Coherence	Adequacy	Relevance	CERQual assessment of confidence in the evidence
Data Culture	[59, 60, 61, 63]	*Minor concerns:* two studies with no concerns, one with minor concerns, and one with moderate concerns. Concerns included details surrounding sampling approach/methods/size and researcher reflexivity.	*Minor concerns:* two studies with no concerns, one with minor concerns, and one with moderate concerns. Concerns included vague and minor references to findings.	*Moderate concerns:* only four studies contributed towards this findings and in some their was only a small inferred reference.	*Moderate concerns:* one study with minor concerns and the remainder had moderate concerns. Concerns included specific settings which narrowed relevance, and also broader aims.	*Low confidence*
**Theme 4: Data Quality**						
Data Accuracy	[55, 57, 58, 59, 44, 60, 61, 45, 62, 63]	*Minor concerns:* four studies had no concerns, three studies had minor concerns, and three had moderate concerns. Concerns included lack of researcher reflexivity, use of broad secondary analysis, and lack of detail surrounding sampling decisions/methods.	*Minor concerns:* seven studies with no concerns, and three with moderate concerns. Concerns included data that only briefly touched upon finding or were more conceptual in inference.	*No concerns:* as present in the majority of studies and in most cases with a large amount of detail.	*Moderate concerns:* two studies with minor concerns, seven studies with moderate concerns, and one with high concern. Concerns included very specific contexts, sample and intervention alongside broader research aims.	*Moderate confidence*
Data Validity	[55, 57, 58, 59, 44, 60, 45, 62, 63]	*Minor concerns:* three studies with no concerns, three studies with minor concerns, and three studies with moderate concerns. Concerns included lack of researcher reflexivity, small sample sizes that lacked detailed explanation, and time frame of data collection.	*No concerns:* in any of the studies that contribute to this finding.	*No concerns:* as finding was found in the majority of studies with good coverage.	*Moderate concerns:* two studies with minor concerns, six studies with moderate concerns, and one study with high concerns. Concerns included broad research aims, and highly specific contexts/sample/interventions.	*High confidence*
**Theme 5: Risks**						
Affective	[55, 57, 59, 44, 60, 45, 63]	*Minor concerns:* two studies with no concerns, three studies with minor concerns, and two with moderate concerns. Concerns included lack of researcher reflexivity, lack of detail on sampling methods/detail, use of broad secondary analysis.	*Minor concerns:* five studies with no concerns, one with minor concerns, and one with moderate concerns. Concerns included differences in description/wording used to outline findings and some detail lacking.	*No concerns:* Majority of studies supported this finding clearly.	*Moderate concerns:* two studies had minor concerns, and five studies had moderate concerns. Concerns included broader research aims and specificity of some studies.	*Moderate confidence*
Behavioural	[55, 58, 59, 44, 60, 45, 62, 63]	*Minor concerns:* two studies with no concerns, three studies with minor concerns, and three studies with moderate concerns. Concerns included lack of research reflexivity, and lack of information about sampling approaches/decisions.	*Minor concerns:* four studies with no concerns, two studies with minor concerns, and two studies with moderate concerns. Concerns included only one respondent referencing finding in one study, and described more conceptually in other studies.	*No concerns:* covered in the majority of studies and in most cases with detail.	*Moderate concerns:* one study had minor concerns, six studies had moderate concerns, and one had high concerns. Concerns included broad research aims and also some very specific study designs.	*Moderate confidence*

Table C1 Quotes that support the results of Theme 1: Data Communication

Theme 1: Data Communication
Presentation<br>*"...some wanted even more granular data so that they could better support improvement in patient outcomes. They also suggested instead of comparing to group mean performance to consider comparing to the top quartile of rheumatologists to better contextualize data and motivate improvements."* (p.920) [55]<br><br>*"To make the reports manageable, some requested only summary information, with the capacity to access more details as their time or interest level increased."* (p.263) [44]<br><br>*"Comments that suggest ways to better present or format the data (eg, separate physician and patient data, place pre and post measures side-by-side), graphs hard to read (too small, too busy)..."* (p.285) [58]<br><br>*"I think it has just way too (much) information, especially NSQIP, [National Surgical Quality Improvement Program] and it's kind of hard to hone in on what it's really saying or what it's telling you ... you know if you want me to look at the report and try to change my behavior I don't really know how I'm going to do that based on the information that I gathered."* (p.643) [63]
Interpretation<br>*"Interpreting the data comprised a substantial part of the interaction between the facilitator and the physicians in the AGF [Audit and Group Feedback] sessions. This process was always present and consisted of cycles of reactions to the data ranging from satisfaction to skepticism; understanding the data in the report through clarifying the findings and questioning the facilitator; justifying or contextualizing the data by trying to identify potential explanations for the findings relating to personal, patient, system, or other factors; and reflecting on the data by sharing with the facilitator and other group members the findings of their own reports and their personal experiences and practices."* (p.4) [56]<br><br>*"I didn't really see anything standing out that I should or I should not be doing because I couldn't really interpret where I was in terms of the kind of data."* (p.639) [63]<br><br>*"I think it's an interesting concept, because I think sometimes we're maybe not the best . . . critics, of ourselves, so having another set of eyes have a look at it is not a bad idea. I think it does depend, of course, on the body that's looking at them and what their expertise is, because, I mean, if it's a group that knows nothing about rheumatology, you know, their opinion may not be as relevant to us as someone who is very well-versed in rheumatology"* (p.919) [55]<br><br>*"...physicians who completed the self-reflection guide reported a need to improve interpretation ability (e.g., the report should summarize three areas where the recipient is doing well and three areas where they can improve as they had difficulty answering this question)."* (p.5) [57]<br><br>*"But if the simple metric is patients per hour then there might be gender affect there that could get interpreted in the wrong way which would say that women should work faster.""* (p.922) [60]

Table C2 Quotes that support the results of Theme 2: Performance Reflection

Theme 2: Performance Reflection
Attribution<br>*"But some of it isn't the doctor's fault that the patient isn't getting seen quickly. It could be availability; it could be booking; it could be patient factors too ..."* (p.920) [55]<br><br>*"Concerns were also raised about being judged on outcomes beyond the physician's control."* (p.263) [44]<br><br>*"The physicians attempted to justify their specified level of performance. The defensive approach was often used when physicians felt there was an external locus of control (or factors outside their control such as non-compliant patients) that impacted their performance."* (p.6) [45]<br><br>*"Although several high volume surgeons were concerned that the data could be compromised by factors outside of their direct control, 1 surgeon thought the variability in personnel should not affect the surgeon's performance and stated, "it's a personal outcome measure that I think is, regardless who is involved, in the end it's assumed to be my responsibility, so it doesn't matter who that team is, the numbers reflect my performance""* (p.641) [63]<br><br>*"It doesn't capture what you're trying to do as a provider. It only captures what the patient decides to follow through on."* (p.5) [57]<br><br>*"I do think it's still helpful to see at the individual level. I think it's more helpful at the home level for sure because I can only impact on those, I can't impact prescribing [of] anyone else."* (p.6) [61]<br><br>*"We have a very high proportion of patients who are under housed, with mental health and addictions and so, there might be a signal there that the reliability of patient data on the quality of care may be influenced by the patient population"* (p.921) [60]
Actionable<br>*"Feedback that provides individual physician data identifying specific improvement areas based on recent patient visits, delivered by an individual that is familiar with the clinical environment, was more favorably received than aggregate-level data based on old data delivered by someone unfamiliar with what physicians deal with day-to-day"* (p.3) [45]<br><br>*"... rheumatologists discussed in what format they would need to see the results for them to be actionable for quality improvement. They regarded the potential to view data longitudinally over time as important to notice trends. While all rheumatologists acknowledged that seeing the data was helpful as they had never had a practice report before, some wanted even more granular data so that they could better support improvement in patient outcomes. They also suggested instead of comparing to group mean performance to consider comparing to the top quartile of rheumatologists to better contextualize data and motivate improvements."* (p.920) [55]<br><br>*"...physicians' intentions when understanding themselves as an Appropriate prescriber was to see how they were doing in comparison to others and decide if they needed to change. The comparison to other physicians in the report data did motivate some to change their practice when the numbers did not meet their expectations..."* (p.6) [61]<br><br>*"They reported that the data could provide valuable insights that could drive positive changes in practice or that the data could be used as a mechanism to monitor changes in practice that were driven by other insights. Physicians in both these groups were keen to engage with the data and explore opportunities for routine use (thereby engaging in the process of reflexive monitoring)."* (p.4) [57]<br><br>*"The comparison to other physicians in the report data did motivate some to change their practice when the numbers did not meet their expectations, such as when they prescribed higher (worse) than average or were at the average but wanted to do better. The intention for change was generally to 'do better' and decrease the initiation and duration of their prescribing, although very few mentioned setting an explicit goal."* (p.6) [61]<br><br>*"Practitioners in the self-data feedback loop are already using the practice data to critically observe their own practice and make determinations on how they may improve."* (p.919) [60]

Table C3 Quotes that support the results of Theme 3: Infrastructure

Theme 3: Infrastructure
Support<br>*"... have a link to resources and or clinical information to support the rational."* (p.288) [58]<br><br>*"Even if enhancing performance is important to physicians, they may be unable to incorporate changes due to time constraints, patient volume and quotas, and not having adequate staff to impact change. Conversely, the presence of a well-staffed and quality team is essential to effect change. Stress, information overload and burnout are also factors that impact the ability to incorporate change."* (p.8) [45]<br><br>*"Some physicians were not deterred, expressing a desire to explore opportunities for further engagement with their data by speaking with a colleague or a similarly trusted source."* (p.5) [57]<br><br>*"The need for support was related to a more deep-seated tension between the desire to provide high-quality patient care alongside system-level pressures, including an ever-increasing number of administrative tasks and numerous QI [Quality Improvement] initiatives. Although physicians described being driven to improve their performance in theory, many were struggling with how to find the time to focus on improvement while simultaneously avoiding burnout."* (p.6) [57]<br><br>*"...physicians rarely had the opportunity to speak to others about their data. One medical director even asked: 'Who am I going to discuss it (the report) with?' 008 (medical director). Some Educators with an interest in discussing and sharing the report had plans to speak to others but had not done so yet, even though most had been receiving the report for several years. One physician (non-medical director) described sharing the data and using the report as a teaching tool with new practitioners, thus filling their role as an Educator and Change driver."* (p.7) [61]<br><br>*Clinicians were most comfortable engaging in A&F [Audit & Feedback] activities within climates of systemic support for improvement. This may include specific coaching constructs around certain elements of clinical practice. Beyond this, some may include short, mid and long-term improvement targets as social contracts with peers.* (p.920) [60]
Culture<br>*"Most surgeons agreed that the report could be reviewed in the group setting, with the potential to identify problems together and discuss ways to improve."* (p.642) [63]<br><br>*"... all surgeons believed that a program of CQI/PD [Continuous Quality Improvement/Positive Deviance] has led to team building through collegial discussions. ... We believe that discussing performance data in a nonthreatening way, colleague to colleague, is an effective method of bringing about change."* (p.1193) [59]<br><br>*"One physician (non-medical director) described sharing the data and using the report as a teaching tool with new practitioners, thus filling their role as an Educator and Change driver."* (p.7) [61]<br><br>*"When systemic supports are in place, they can potentiate and augment the learning that may come from a single individual looking into their own data. Clinicians were most comfortable engaging in A&F activities within climates of systemic support for improvement."* (p.929) [60]

Table C4 Quotes that support the results of Theme 4: Data Quality

Theme 4: Data Quality
Data Accuracy<br>*"The data is only as accurate those who are entering it. I found it interesting, but not sure about the accuracy."* (p.287) [58]<br><br>*"... reasons given for distrust of feedback data included respondent uncertainty about the origin of the data (despite this information being provided at study initiation and in each feedback report); belief that patient encounters were improperly coded or documented in the EHR [Electronic Health Record] ..."* (p.73) [62]<br><br>*"I'm convinced our numbers were skewed. ... we weren't quite sure how they were assembling their data, where they got the numbers from."* (p.76) [62]<br><br>*"Participants' perception of data accuracy led them to report it did not inform their reflections about their practice, which was further reflected in responses to the self-reflection guide. When responding to the question "Please comment on what can be done to make this data and feedback process more useful for you" ... seven physicians suggested a need to improve the accuracy of the data while five provided general feedback to improve the report."* (p.5) [57]<br><br>*"Physicians typically trusted the source of the data, although some needed to work through a process first, such as reading through how they came to the numbers, the source of the original data and so on."* (p.6) [61]<br><br>*"...when the data are of a poor quality, rejecting the data (and calling into question its validity) is a ubiquitous reaction to the provision of poor-quality practice data."* (919) [60]
Data Validity<br>*"Perceived limitations of SSORs [Surgeon-Specific Outcome Reports] included ... inaccurate representation of performance ..."* (p.1191) [59]<br><br>*"The reasons given for distrust of feedback data included ... belief that the audit could not capture the complexity of a patient's situation ..."* (p.73) [62]<br><br>*"... limitations included inappropriate comparisons between surgeons ..."* (p.64) [63]<br><br>*"Some thought if the data were more representative of their whole practice (say from an EMR [Electronic Medical Records]) that they might have greater incentive to make practice changes."* (p.921) [55]<br><br>*"When they feel performance is based on a small sample of patients that is not representative of the care they provide they ignore the feedback and do not take any action. ... "The N is incredibly tiny. These patients may not be representative of our typical patient, yet these numbers are taken very seriously.""* (p.6) [45]<br><br>*"... may not be representative of rheumatologists' practices causing concern about generalizability of results ". . . so I don't know how valid is that, how far can you take it""* (p.919) [55]<br><br>*"Most participants felt that the data inadequately reflected the complexities of patient care, including the element of patient choice."* (p.4) [57]<br><br>*"Questioning data validity occurred during the discussion of many areas of data feedback although were particularly relevant in questions centring around patient satisfaction and teaching evaluations by learners."* (p.921) [60]

Table C5 that support the results of Theme 5: Risks

Theme 4: Risks
Affective Risks<br>*"Surgeons also feared that sensitive data within the SSORs [Surgeon-Specific Outcome Reports] could be used for punitive actions ..."* (p.1192) [59]<br><br>*"It wanted me to sign that I was going to improve x, x, and x, over the next period of time and so on. I just said, "No way, I am not buying into this guilt trip." I tried and I'm not perfect. I'm going to continue to try but I'm not going to be burdened with extra guilt."* (p.262) [44]<br><br>*"Negative emotion is often coupled with nonacceptance and results in no behavior modification. When physicians are irritated, frustrated or become apathetic over the assessment process change is less likely."* (p.8) [45]<br><br>*"Participant narratives revealed that this tension was exacerbated by a pre-existing apprehension and uncertainty around a broader, system-level movement towards accountability. Participants who were anxious about data being used for accountability were initially reticent to trust the data, an emotional response that needed to be addressed to support meaningful action."* (p.6) [57]<br><br>*"Many clinicians exhibited feelings of being overwhelmed with the data or feeling helpless as a result of receiving data. Many explicitly endorsed a devaluation of self-image and stated that it could be difficult to face their perceived shortcomings before moving to an exploration of practice improvement. Some respondents identified that it may be easier to not be made aware of opportunities for improvement."* (p.922) [60]
Behavioural Risks<br>*"Most surgeons thought the impact of taking care of high-risk patients would worsen their SSR [Surgeon-Specific Reports] metrics. One surgeon referenced the cardiac surgery experience with individual outcomes. He/she described how physicians would "cherry-pick" their patients, likely in an attempt to keep "excellent outcomes.""* (p.641) [63]<br><br>*"People may get the wrong impressions and some people would then, as a remedy for this, instead of fixing the problem, go try to fix the report."* (p.642) [63]<br><br>*"Practice change for some was unlikely "I don't think it's going to change anything else, to be honest""* (p.920) [55]<br><br>*"Others raised important insights into the consequences of data feedback that may be viewed as negative by the recipient. They highlighted that the risk of these could be inappropriate remedial actions to improve data metrics or that the data may be seen as a value judgement on their character."* (p.921) [60]
